# Host-Microbiome Interactions in the Era of Single-Cell Biology

**DOI:** 10.3389/fcimb.2020.569070

**Published:** 2020-10-14

**Authors:** Prateek V. Sharma, Christoph A. Thaiss

**Affiliations:** Microbiology Department, Perelman School of Medicine, University of Pennsylvania, Philadelphia, PA, United States

**Keywords:** microbiome, single-cell sequencing, genomics, host-microbiome interaction, technology, microbial heterogeneity

## Abstract

Microbes are the most prevalent form of life yet also the least well-understood in terms of their diversity. Due to a greater appreciation of their role in modulating host physiology, microbes have come to the forefront of biological investigation of human health and disease. Despite this, capturing the heterogeneity of microbes, and that of the host responses they induce, has been challenging due to the bulk methods of nucleic acid and cellular analysis. One of the greatest recent advancements in our understanding of complex organisms has happened in the field of single-cell analysis through genomics, transcriptomics, and spatial resolution. While significantly advancing our understanding of host biology, these techniques have only recently been applied to microbial systems to shed light on their diversity as well as interactions with host cells in both commensal and pathogenic contexts. In this review, we highlight emerging technologies that are poised to provide key insights into understanding how microbe heterogeneity can be studied. We then take a detailed look into how host single-cell analysis has uncovered the impact of microbes on host heterogeneity and the effect of host biology on microorganisms. Most of these insights would have been challenging, and in some cases impossible, without the advent of single-cell analysis, suggesting the importance of the single-cell paradigm for progressing the microbiology field forward through a host-microbiome perspective and applying these insights to better understand and treat human disease.

## Introduction

Microbial organisms are the predominant life form inhabiting our planet, with approximately 10^30^ cells of bacteria and archaea estimated to exist on Earth. A recent scaling estimate placed Earth's inhabited microbial species count at 10^11^–10^12^ (Locey and Lennon, 2016). Despite the great magnitude of ecological diversity and prevalence, microbes remain some of the least characterized organisms, with potentially more than 99% of microbial taxa remaining to be discovered (Locey and Lennon, 2016). With each human estimated to host 10^13^–10^15^ microbial cells, a count as large as the number of somatic cells in our bodies, it is undeniable that a more complete appreciation of the human microbiome also yields a greater understanding of human health and disease (Sender et al., 2016; Gilbert et al., 2018). Indeed, many investigations in the past decade have implicated the microbiome in a variety of human disorders, including inflammatory bowel disease (Frank et al., 2007; Gevers et al., 2014; Thaiss et al., 2016b), cancer (Pleguezuelos-Manzano et al., 2020), CNS disorders (Jiang et al., 2015; Keshavarzian et al., 2015; Zheng et al., 2016), cardiovascular disease (Jie et al., 2017), and obesity (Le Chatelier et al., 2013; Goodrich et al., 2014; Thaiss et al., 2016a; Thaiss, 2018).

Over the past few decades, technological advancements in sequencing and model systems for studying the microbiome have tremendously benefited research seeking to better characterize its role in organism development and disease pathology. The germ-free humanized mouse became broadly adopted in the field as a method to study the microbiome in a model organism (Samuel and Gordon, 2006). Meanwhile, at the genomic level, the most commonly used methods for characterization include 16S ribosomal DNA (rDNA) sequencing, metagenomic and metatranscriptomic sequencing, and metabolomic characterization of the microbiome to better understand composition and colony-level features (Tolonen and Xavier, 2017).

Despite this progress, there are challenges to understanding microbiome heterogeneity, though its importance has long been appreciated in the context of phenotypically variable diseases such as IBD (Sun et al., 2019). In particular, commonly used methods often lose spatial and cellular stratification of microbes across colonies and within species (Hatzenpichler et al., 2020). Recent advancements in single cell isolation and sequencing technologies offer a potential solution to the technically limited analysis of microbial heterogeneity (Blainey, 2013). However, several factors impede characterization of microbes by traditional single-cell sequencing methods. Low DNA and mRNA content limit the yield of reasonable amounts of genetic material for sequencing analysis from a single cell. The lack of polyadenylation of bacterial mRNA limits its separation from rRNA. Additionally, the diversity of cell walls and membranes poses a challenge to consistent lysis or permeabilization required for single-cell RNA sequencing (scRNA-seq).

Several techniques have begun to address the aforementioned limitations. Fluorescence-activated cell sorting (FACS) has been applied to uncultivated microorganisms to achieve single cell isolation followed by lysis, whole genome amplification (WGA), and 16S rRNA-based identification of cells (Rinke et al., 2014). The application of microfluidics to cell isolation has rapidly grown in use within the field of microbiology, enabling high-throughput isolation, fragmentation, and barcoding of single-cell microbial genomes (Lan et al., 2017). Single droplet multiple displacement amplification (sd-MDA) captures single cells in picoliter droplets followed by whole genome amplification, preserving the integrity of single genome specificity (Hosokawa et al., 2017). However, a limitation of this approach is that MDA can amplify DNA contamination, yield uneven read coverage, and lead to chimera reads that link non-adjacent template sequences (Zhang et al., 2006). Gel microdroplet cultivation is a method in which single cells are captured in agar droplets and grown to a population of hundreds of cells before MDA (Fitzsimons et al., 2013). While this allows for amplification of genomes from single cells, this can yield sampling bias based on the requirement for cell cultivation in agar (Tolonen and Xavier, 2017).

In the following, we highlight the emerging technologies used to better characterize both the innate human microbiome as well as host-microbiome relationships at single-cell resolution. These advancements can be classified as those pertaining to the genomic and transcriptomic diversity at the cellular level in the microbiome as well as the spatial distribution of microbes conferring heterogeneity at the colony level.

## Microbiome Studies at Single-Cell Resolution

### Resolving Taxonomic and Functional Heterogeneity

**Figure 1:**
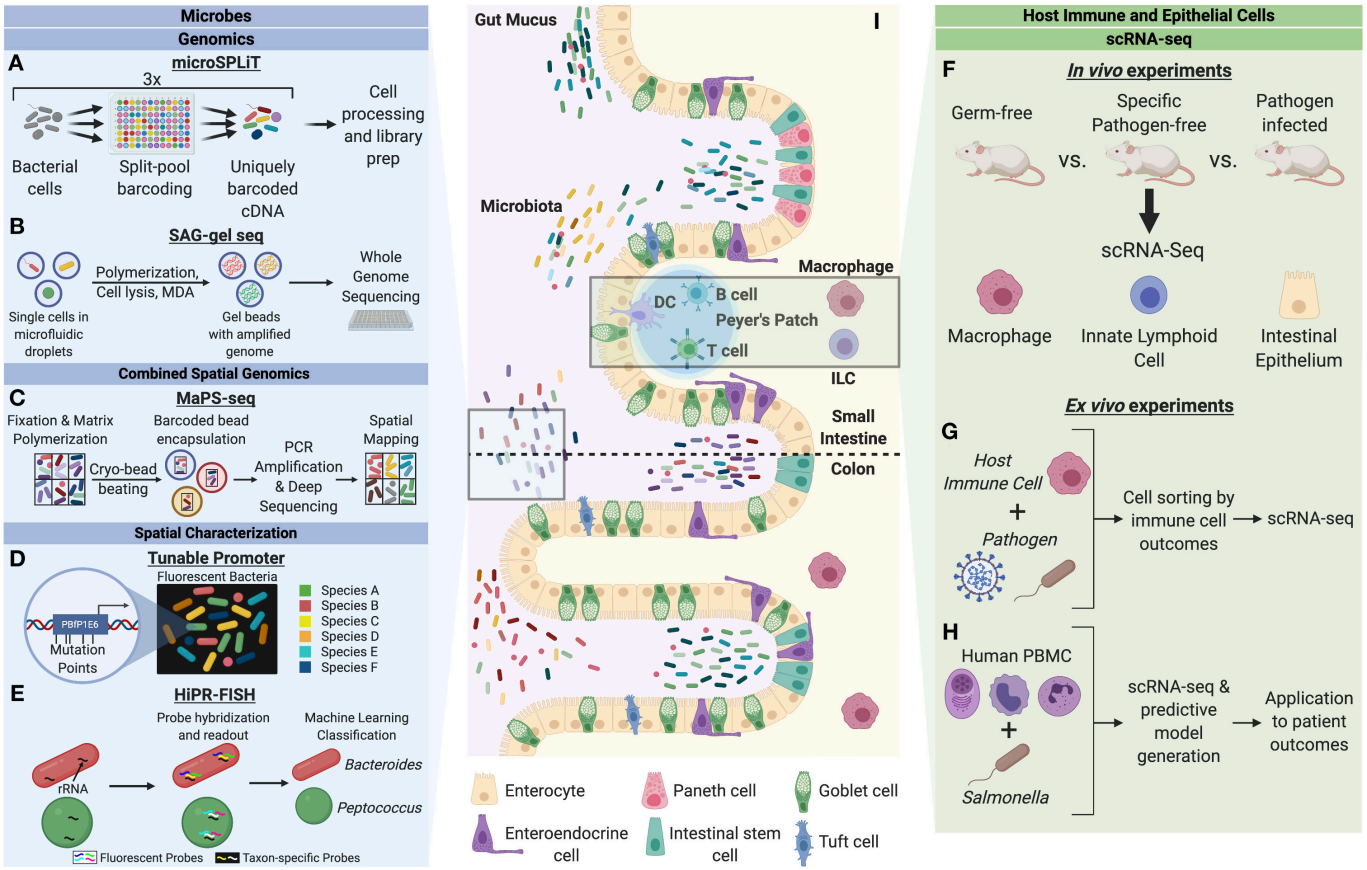
Single-cell characterization techniques for microbes and host cells involved in microbial interactions. Microbes can be studied through a variety of single-cell genomics, spatial characterization, and combined spatial genomic techniques. **(A)** Microbial split-pool ligation transcriptomics (microSPLiT) leverages multiple rounds of cell pooling and random splitting to uniquely barcode cDNA by cell of origin, leading to unbiased capture of RNA expression profiles and bypassing a requirement for single cell isolation (Kuchina et al., 2019). **(B)** Single amplified genome (SAG) gel sequencing involves single bacterial cell isolation via microfluidic droplets, two rounds of parallel multiple displacement amplification (MDA), and multiplex single-cell genome sequencing (Chijiiwa et al., 2020). **(C)** The combination of spatial and genomic resolution is leveraged in metagenomic plot sampling by sequencing (MaPS-seq) (Sheth et al., 2019). Input microbiome samples are fixed in a gel matrix and cryofractured to yield spatial clusters, which can be encapsulated by barcoded beads and subjected to 16S rRNA amplification and deep sequencing. **(D)** The tunable promoter technology allows for generation of promoters with expression levels across a broad range. This can be applied to create species-specific fluorescence signatures to visualize strain-level distinction of bacterial cells *in vivo* (Whitaker et al., 2017). **(E)** Highly phylogenetic resolution fluorescence *in-situ* hybridization (HiPR-FISH) relies on a binary system of taxa barcoding based on hybridization of up to 10 distinct fluorophores (Shi et al., 2019). The barcode spectra are decoded via a machine-learning classifier to allow for taxa identification and visualization. Host cells can be studied through *in vivo* and *ex vivo* mechanism to understand cell heterogeneity in the context of microbial influences. **(F)** A variety of host cells can be studied through single-cell RNA-seq (scRNA-seq) of cells from model organisms with varying degrees of microbiota alteration (Gury-BenAri et al., 2016; Kang et al., 2020). **(G)** Host immune cells subjected to pathogens can be sorted by cell outcomes and characterized by scRNA-seq to understand host heterogeneity in the context of pathogenic microbes (Avraham et al., 2015; Saliba et al., 2016). **(H)** Human peripheral blood mononuclear cells (PBMC) can be studied in response to pathogenic microbes to generate predictive models of disease outcomes for patients (Bossel Ben-Moshe et al., 2019). **(I)** The intestinal epithelium is a classic example of a host-microbiota interface. Single-cell analysis of microbial and host cells offers insight into their biological heterogeneity as well as the influence of each organism on adaptive cellular responses by the other.

Microbial single-cell genomics is a very recent and rapidly emerging field. Advances in single-cell genomics developed for eukaryotic cells have enabled the recent development of tools that can likewise be applied to prokaryotes. One such technique, termed SPLiT-Seq, involves combinatorial barcoding of RNA and has yielded novel insights into bacterial transcriptomics at the single-cell level. In SPLiT-Seq, cells are fixed, permeabilized, and cDNA is generated from cellular RNA through intracellular reverse transcription (RT) with barcoded poly-T and random hexamer primers in a multi-well format (Rosenberg et al., 2018; Figure 1A). Multiple rounds of cell pooling and random splitting followed by well-specific cDNA barcoding ensure a high likelihood of unique tagging of RNA per cell origin. This technique is well-suited to microbial application due to its ability to bypass single cell isolation and allow unbiased capture of RNA expression profiles due to the random hexamer capture of transcripts. To achieve mRNA enrichment, a recent study utilized *Escherichia coli* Poly(A) Polymerase I (PAP) to preferentially polyadenylate mRNA in cells (Kuchina et al., 2019). This study in particular applied split-pooling, termed “microSPLiT,” to identify a variety of bacterial subpopulations with differential gene expression patterns across a range of stress responses, metabolic pathways, and bacterial growth and development (Kuchina et al., 2019). Through analysis of heat shock exposure in *Escherichia coli* MW1255 and *Bacillus subtilis* PY79 cells, temporal activation of housekeeping and stress response sigma factors was identified, organized into sub-clusters of bacterial populations. Further analysis revealed temporal changes in regulation of carbon utilization, stress responses, metal uptake, and developmental decisions along growth phases, indicating subpopulation heterogeneity across a wide range of pathways. Additionally, another key finding was the enrichment in competent state transcriptional signatures upon later phases of bacterial growth curves.

A similar technique employing split-pool sequencing has also been developed, referred to as Prokaryotic Expression-profiling by Tagging RNA *in situ* and sequencing (PETRI-seq) (Blattman et al., 2020). PETRI-seq consists of three main components: cell preparation, split-pool barcoding, and library preparation. A few notable differences in the protocol from microSPLiT include lack of preferential mRNA capture and substitution of streptavidin capture for cDNA purification with the use of AMPure XP beads to purify cDNA from cell lysates. PETRI-seq was able to robustly discriminate single *E. coli* cells by different phases of growth, finding expected trends for expression of stationary-phase associated genes, ribosomal protein expression, and amino acid biosynthesis. Using PETRI-seq, the authors applied principal component analysis to 6,663 *Staphylococcus aureus* single-cell transcriptomes and were able to detect a rare subpopulation (0.04%) of cells undergoing prophage induction, enriched for lytic genes of prophage φSA3usa.

Another technique that has recently been applied to microbial analysis is Single Amplified Genome (SAG) sequencing (Chijiiwa et al., 2020; Figure 1B). A specific variant of SAG sequencing termed SAG-gel involves three steps: single bacterial cell isolation with gel beads, two rounds of parallel multiple displacement amplification (MDA), and multiplex single genome sequencing. The first step is accomplished through microfluidic droplet generators for single cell encapsulation in an agarose solution. Upon chilling of the single-bacteria containing droplets, the agarose polymerizes to form beads which are then subjected to cell lysis reagents and MDA. Gel beads positive for amplification of genome, identified by SYBR Green staining, are then sorted into 96-well plates and subjected to a second round of MDA. Following quality control steps for DNA yield and contamination, whole genome sequencing is performed. One study examined the effects of dietary fiber inulin on the composition of gut microbiota in mice (Chijiiwa et al., 2020). Using 16S rRNA gene sequencing to initially categorize bacterial responders at the family level, SAG sequencing was then applied to mouse gut microbiota to understand inulin utilization abilities of specific bacteria that were increased upon inulin feeding. A key finding from this study was the identification of two responder Bacteroides genomes with polysaccharide utilizing loci clusters containing inulinases. These genomes were similar to known inulin utilizers and considered a potential new subgroup with *B. acidifaciens*. In addition, further genomic analysis of these two responder Bacteroides compared to non-responders showed differences in conserved biosynthetic pathways in cofactor and vitamin metabolism, amino acid transport and biosynthetic pathways, and carbohydrate metabolism, suggesting that inulin responders play different metabolic roles in the mouse gut microbiota.

To address biased genomic coverage and chimeric sequences found in single-amplified genomes, one study developed a novel analytical workflow termed Cleaning and Co-assembly of a Single-Cell Amplified Genome (ccSAG) (Kogawa et al., 2018). In ccSAG, raw SAGs are first grouped by identical microbe strains based on 16S rRNA similarity in the V3-V4 region and average nucleotide identity. Raw contigs are constructed from these SAGs and compared within group and classified as clean, unmapped, or potentially chimeric reads. Chimeras are split based on alignment to raw contigs and remapped. Cycles of cross-reference mapping and chimera splitting ultimately identify unmapped reads and remove chimeras. Finally, clean reads are co-assembled *de novo* into composite SAG contigs and bridged using raw composite SAG contigs. This yields gap-free composite single-cell genomes. This analytical process was applied to microfluidic-based single-cell MDA sequencing of mouse gut microbes (Kogawa et al., 2018). From this study, two novel draft genomes within the Bacteroides strain were identified with differences in metabolic pathways, such as cobalamin biosynthesis, suggesting distinct metabolic roles for these novel strains. Of note, when coding sequences from SAGs within strains were compared, single nucleotide polymorphisms (SNPs) that resulted in an amino acid change were detected within a polysaccharide lyase gene. Whereas conventional SAG co-assembly may overlook such SNPs due to the construction of composite single-cell genomes, the analytical method of ccSAG allowed for detection of within-strain SNPs that suggest greater genetic and functional heterogeneity within the same microbial species than previously appreciated.

In contrast to techniques of flow cytometry and traditional microfluidic-based sequencing, virtual microfluidics was developed whereby instead of physical microfluidic compartmentalization to isolate single molecules and cells, a bulk polyethylene glycol (PEG) hydrogel is utilized to achieve diffusion-based compartmentalization without discrete borders between molecules or cells (Xu et al., 2016). In this format, small molecules and oligonucleotides can freely move between virtual “compartments” while single cells and high-molecular-weight MDA products cannot. This format allows for easy physical access to single cells and MDA product clusters for imaging, analysis, and localized barcoding. Moreover, the local restriction of MDA intermediates can address technical limitations like chimeric reads, increasing the integrity of single-cell MDA products at larger scales. This technology was tested for single-cell shotgun genome sequencing of mixed cultures of *E. coli* and *S. aureus*, resulting in successful resolution of single-cell amplification products with no cross-contamination (Xu et al., 2016). In the same study, this technique was applied to diverse and uncharacterized microbial species in human stool samples from the Fiji Community Microbiome Project. Virtual microfluidics identified a microbial family not initially detected in standard taxonomic assignment of shotgun data. This suggests the importance of an unbiased single-cell approach like virtual microfluidics for microbe analysis, especially when particular organisms might not be well represented in reference datasets.

The findings from each of these approaches are examples of fundamental discoveries regarding microbial properties that would otherwise have been missed in bulk sequencing, which limits detection of cell-to-cell variability that is often critical in allowing particular sub-populations to arise upon environmental changes. The ability to obtain single-cell genomic resolution has important clinical implications, such as the characterization of bacterial persistence and unculturable constituents of large microbial communities. Additionally, these new technologies preclude the need for reference genomes. This allows for the analysis of uncultured bacteria from the host, many of which have not yet been cataloged or characterized, and opens new insights into the microbiome composition.

### Resolving Spatial Heterogeneity

With the advantage that genomic sequencing offers for better deconvolution of complex microbial subpopulations at the single-cell level comes a drawback of the loss of spatial information that such complex populations contain. Currently established methods of visual imaging pose a challenge of strain-level differentiation in mixed communities. On this front, several studies have looked at providing single-cell spatial information on microbial communities.

One example is an AT-rich ribosome binding site (RBS) library resembling the residues found upstream of *Bacteroides fragilis* (Bf) phage genes, from which the highest expression producing promoter sequence was identified, termed PBfP1E6 (Whitaker et al., 2017). In Bacteroides strains, PBfP1E6-driven expression exhibits at least an order of magnitude greater fluorescence compared to commonly used promoters and 70-fold higher than the 16S rRNA promoter. Notably, this promoter did not detectably reduce fitness of bacterial strains *in vivo*. Through mutational analysis, a series of interchangeable promoters of varying strengths was created spanning a 30,000-fold expression range. Six different Bacteroides species were engineered to produce unique fluorescent signatures based on combinations of different levels of GFP and mCherry expression (Whitaker et al., 2017; Figure 1D). These were able to be identified *in vivo* with a low cell identification error (~6%). As a result of this technology, the authors sought to examine the influence of crypt colonization of the mouse gut by Bacteroides on subsequent colonization attempts of an isogenic strain. Crypt association had been suggested to be important in maintaining colonization, however this particular study allowed for spatial resolution of isogenic strain localization. Notably, sequential colonization resulted in the second strain exhibiting significantly less overall colonization, which was greatly exaggerated at the crypt relative to the lumen, providing additional evidence that crypt colonization plays an important role in Bacteroides entrenchment in the gut. This study is an early example of how such molecular tools can be leveraged to provide strain-level resolution to uncover colonization heterogeneity of resident gut microbes.

Fluorescence *in-situ* hybridization (FISH) assays that target rRNA for taxonomic identification and visualization currently exist but are limited in taxonomic resolution. One modification of this fluorescence-based assay that has recently been developed is High Phylogenetic Resolution FISH (HiPR-FISH) (Shi et al., 2019; Figure 1E). This technique offers high multiplexity via a binary system of barcodes using a two-step process: the first step takes advantage of taxon-specific 16S rRNA probes while the second step involves hybridization with a cohort of fluorescently labeled readout probes. The uncoupling of taxon-specific 16S rRNA probes with fluorescent readout enables scaling of this technique to sufficiently large numbers of unique combinations to study complex microbial ecosystems in nature. To achieve single-cell quantitation, the authors modified existing algorithms for automated image segmentation for single cells after HiPR-FISH followed by rounds of pixel classifications and filtering for image optimization. This method enabled the quantitative physical analysis of microbial species in the human oral plaque microbiome (Shi et al., 2019). Specifically, novel micro-architectures of cells from particular genera previously undescribed in imaging experiments—likely due to their low prevalence—were able to be appreciated from this work. In the application to the mouse gut microbiome, HiPR-FISH maps were created for mice treated with two different antibiotics and controls. The authors found that antibiotic treatment led to particular alterations to the spatial organization of adjacent taxa in the mouse gut, showing the importance of considering relative spatial biogeography in microbiome analysis, in addition to relative species abundance. Unique advantages of HiPR-FISH include the requirement of only a single round of imaging to decode multi-color barcodes *in-situ*, resulting in faster data acquisition than traditional FISH technologies. Moreover, the ability to multiplex with multiple rounds of hybridization and imaging allows for further separation of initial readouts.

Given the newly developed ability to appreciate the microbiome at both the genomic and spatial levels, the natural progression is to identify how these two critical sources of information can be tied together to offer spatial genomic analysis of the microbiome. To this end, a method of metagenomic plot sampling by sequencing (MaPS-seq) was developed that preserves microbial cells in their native biogeographical context to create a spatial map of the microbiome (Sheth et al., 2019; Figure 1C). MaPS-seq begins by fixation of an input tissue sample that is then permeabilized and incubated with an acrylamide solution containing reverse 16S rRNA amplification primers. This sample is then fractured via cryo-bead beating to generate cell clusters, subjected to cell lysis, and filtered through nylon mesh for particle size selection. These resulting clusters contain genomic DNA in a geographically preserved fashion, enabling capture of spatial information. These clusters are then co-encapsulated with gel beads containing uniquely barcoded forward 16S rRNA amplification primers which can be photocleaved from the beads to ensure release of genomic DNA upon polymer matrix degradation. The resulting droplets are subjected to PCR amplification followed by droplet separation and deep sequencing. These sequencing reads are grouped by unique barcodes and subsequently categorized by relative abundance of bacterial operational taxonomic units (OTUs). The authors applied MaPS-seq to study the mouse gut microbiome along different regions of the GI tract, specifically the ileum, cecum, and distal colon (Sheth et al., 2019). They observed differences in taxonomic association when comparing GI sites, however some common co-associations were observed, such as a positive association between *Lachnospiraceae* and *Lactobacillaceae* found in both the cecum and colon. These spatial architectures suggest that although environmental factors can variably shape local spatial structuring of the microbiota there are more robust associations not affected by environmental variation. Moreover, when applied to studying the spatial organization of distal colon microbiota upon diet changes, MaPS-seq revealed particular phylogenetic clustering of taxa in low-fat, plant-polysaccharide based diet that were not observed in mice given high-fat, high-sugar diets (Sheth et al., 2019). Upon t-SNE analysis, clusters from these two diets formed highly distinct groups with limited overlap, indicating a substantial change in spatial organization upon the dietary shift. The technique of MaPS-seq provides a new level of insight into the spatial organization of microbial taxa across different host environments and in the context of environmental perturbations that would have been overlooked through conventional approaches.

Together, these studies show the inherent power of complementing currently available metagenomic analysis with single-cell genomics and spatial characterization. The combination of these analyses may ultimately enable a characterization of microbial biogeography that has been underappreciated and unravel the complexities inherent to the host microbiome along with the impact of environmental influences on the spatial relation between and genomic changes to microbes in real time.

## Single-Cell Studies of Host Heterogeneity in the Context of Microbes

Acknowledging the taxonomic and functional heterogeneity in microbial communities, it is particularly interesting to examine the degree of adaptation and variability of host responses to microbial challenges. Analysis of microbes at the single-cell level can not only provide insight into the microbiome, but offer the potential to better characterize the intercellular relationship between microbes and their hosts. This is fundamental to the context of physiology and pathophysiology, environmental influences on host immunology, and the role of host cells in modulating the microbiome.

### Host Interactions With Commensals

Single-cell analysis of host cells has provided novel insight into the role of both commensal and pathogenic microbes in modulating host physiology. One study in particular performed single-cell RNA sequencing on colon macrophages of germ-free (GF) and specific pathogen-free (SPF) mice (Kang et al., 2020). When comparing all clusters of colon macrophages from SPF mice to GF, the SPF macrophages were found to have an increase in expression of genes associated with immune defense, antigen presentation, and oxidative phosphorylation. At the cluster-level analysis, two particular macrophage clusters exhibited a marked increase in SPF mice: the first being CD11c^+^CD206^int^CD121b^+^, which displayed high expression levels of genes involved in antigen processing and presentation, lipid localization, and cell migration, and the second cluster being CD11c^−^CD206^hi^CD121b^−^, which displayed increase in genes involved in the response to interleukin-1, wound healing, vasculature regulation, apoptotic cell clearance, and cytokine production. The germ-free mice displayed enrichment in a population of macrophages with lower gene expression for inflammatory and stress responses. These two clusters were found to derive from a common precursor cluster of macrophages with high CCR2 expression levels. A loss of CCR2 along with increases in pan-macrophage markers in the CD11c^+^CD206^int^CD121b^+^ and CD11c^−^CD206^hi^CD121b^−^ cells followed a pseudo-time course. This work was validated through flow cytometry of the distinct subpopulations of colon MPs with bulk RNA sequencing analysis. Thus, the application of scRNA-seq to study host cells at the cellular level has identified the generation of particular colonic macrophage subpopulations that are dependent upon a bacteria-driven differentiation trajectory in the host gut (Figure 1F).

Innate lymphoid cells (ILC) are the most recently discovered component of the innate immune system and serve as key modulators of mucosal immunity, inflammation, and tissue homeostasis (Xu and Di Santo, 2013; Artis and Spits, 2015). Helper-like ILCs can be subdivided into three distinct types based on differential expression of transcription factors that lend diverse characteristics to ILCs. To determine the response to ILCs to the microbiome, and subsequently how changes in the microbiome alter ILC biology, one study applied scRNA-seq to uncover the response of ILCs to microbial colonization at the single-cell level (Gury-BenAri et al., 2016). Analyzing the small intestinal mucosa of antibiotic-treated and GF mice, the authors found a similar profile of clusters for antibiotic-treated and GF mice, both of which were substantially distinct from SPF mice, suggestive of a similar effect of pre- or post-development microbiota depletion on ILCs. In both antibiotic-treated and GF mice, when comparing relative abundances of the ILC subgroups, there was an observed expansion of ILC3 and ILC2 cells with loss of ILC1 phenotypes. In the ILC1 and ILC2 subpopulations, a sharp loss of expression was observed in ILC2-specific genes alongside increases in ILC3-specific genes. Additionally, the expression of cytokine IL-17a, previously understood to be dependent on the microbiome, was consistently lost across all subgroups in antibiotics-treated and GF mice, adding a further line of evidence to the microbiome impact on IL-17a expression. Ultimately, the application of scRNA-seq to immune cells that are in close cellular proximity to the microbiome unveiled cellular level changes in identity and cell fate regulation that would otherwise have been lost in bulk sequencing.

### Host Interactions With Pathogens

In addition to commensals, pathogenic microorganisms likewise show a high degree of within-population heterogeneity in composition and function (Balaban et al., 2004; Stewart et al., 2011; Claudi et al., 2014). This raises the question of how cell-to-cell variability predicts or alters the host relationship with microbial organisms in a pathological context. This has been explored in studies of *Salmonella* Typhimurium infection of macrophages to uncover the underlying mechanism of the complex, heterogenous immune response to infectious microbes (Figure 1G). In recent years, much insight has been gained into host responses to microbial infections, which has served as a valuable tool for elucidating host immune-microbiome relationships at the single-cell level (Chattopadhyay et al., 2018).

As macrophages exhibit variable outcomes in response to pathogenic microbes—no infection, infection with pathogen destruction, infection with pathogen persistence—one study performed scRNA-seq on *Salmonella*-exposed mouse bone marrow-derived macrophages (BMMs) to distinguish transcriptional changes alongside immunological response outcomes (Avraham et al., 2015). Principle component analysis revealed gene clusters that stratified macrophages by exposure and non-exposure to pathogen as well as by infected and uninfected macrophages. One gene cluster enriched for type I interferon (IFN) response, however, was induced in a subset of macrophages and found to distinguish infected from uninfected, yet pathogen exposed, macrophages. Among the sub-population of infected macrophages, genes primarily responsive to intracellular signals of infection were more variable than those responsive to extracellular cues of bacterial expression. This difference suggests the inherent heterogeneity even among phenotypically homogenous populations of infected cells. Upon further investigation of infected macrophages, the expression level of *Salmonella* virulence factor PhoPQ, known to upregulate genes important for intramacrophage survival, was found to determine the level of type I IFN induction in infected macrophages. In this study, single-cell analysis was critical in the identification of such a population as the type I IFN gene cluster was not highly induced when averaging over all cells. This work highlights the importance of pathogenic variability and host response heterogeneity of infected and bystander cells, suggesting that the state of immune activation does not operate in a silo of the host, but also mirrors the intrinsic variation found in pathogens that subsequently mold the host response.

Expanding upon this study, another group sought to explore how macrophages respond to extremes of intracellular bacterial growth heterogeneity. Through fluorescent *Salmonella* strains reporting bacterial proliferation inside mouse BMMs, the authors combined cell sorting and scRNA-seq and identified three subpopulations of macrophages: naïve macrophages, and two groups of challenged macrophages (Saliba et al., 2016). Upon analysis of these two challenged macrophage groups, the expression profiles of macrophages containing non-growing bacteria were found to display hallmarks of the pro-inflammatory M1 polarization state and were indistinguishable from those of bystander cells, suggesting that non-growing bacteria evade intracellular immune receptor recognition. By contrast, macrophages containing growing bacteria assumed an anti-inflammatory, M2-like state, suggesting that rapidly growing *Salmonella* reprogram macrophage polarization to avoid host responses. This was found to be a direct result of infection, as such M2 markers were absent from naïve macrophages and shown to be correlated with bacterial proliferation. Moreover, the authors also identified a range of intermediate states between these two macrophage extremes. This study demonstrates the recently appreciated concept that microbes can leverage host genome plasticity to accomplish their own biological requirements of maintenance or proliferation.

Combining the sequencing of macrophages and prokaryotic pathogens, another study applied scRNA-Seq to *Salmonella*-infected macrophages to perform a dual analysis of both host and pathogen in a single-cell context, termed scDual-Seq (Avital et al., 2017). This method involves the priming of reverse transcription via random hexamer DNA oligos, the use of barcoded primers for multiplexing, *in vitro* transcription for RNA amplification, and paired-end Illumina sequencing. Reads are then mapped to the mouse and *Salmonella* transcriptomes. Through this study, the authors identified two classes of intracellular *Salmonella* with distinct transcriptional signatures denoted as Class I and Class II. Moreover, they found three subpopulations of infected macrophages: partially induced macrophages infected with Class-I-like *Salmonella*, fully induced macrophages infected with Class I *Salmonella*, and fully induced macrophages infected with Class II *Salmonella*. Through pseudo-time analysis, this study found macrophages to follow a linear progression from the partially induced state to the fully induced state alongside changes in the *Salmonella* Class from I to II. This study represents a new paradigm with which to study host-pathogen interactions through simultaneous analysis of both transcriptomes. Despite limitations in phenotype resolution due to variable multiplicity of infections, future work can continue to build off of this method of linked analysis of host and pathogen.

In addition to immune cells, epithelial cell responses to pathogens have also been shown to play vital roles in host homeostasis. One study examined the effect of *Salmonella* and the helminth *Heligmosomoides polygyrus* on host intestinal epithelial cells (Haber et al., 2017). Through the technique of droplet-based scRNA-seq, the authors profiled epithelial cells from the small intestine of mice after different durations of infection. The responses to pathogens were sorted into pathogen-specific and pathogen-shared cell-intrinsic changes and shifts in intestinal cell composition. In terms of cell-intrinsic responses to *Salmonella*, expression shifts were observed across all infected epithelial cells in genes involved in pathways for defense responses to bacteria. Interestingly, some responses to *Salmonella* were found to be induced in a cell-type specific manner, such as various antimicrobial peptides and pro-inflammatory proteins. Conversely, other proteins that were previously thought to be cell-type specific, such as the anti-microbial peptide *Reg3a*, were found in all cell types post-*Salmonella* infection. In response to helminth infection, most induced genes were pathogen-specific genes and included inflammatory-response genes and tuft cell markers. Within goblet cells, genes previously implicated in antiparasitic immunity were found to be induced. Of note, some genes identified in the goblet cell *H. polygyrus* response (*Wars* and *Pnlipr2*) were not previously known to be expressed in these cells. Both *H. polygyrus* and *Salmonella* infections resulted in upregulation of stress gene modules in stem cells. In terms of cell composition changes, *Salmonella* infection led to increases in mature enterocytes and Paneth cells along with significant reductions in transit-amplifying and stem cells. *H. polygyrus* infection substantially increased goblet cell and tuft cell count along with reductions in enterocytes. These results suggest that a more complete understanding of the host response to pathogens involves a mixture of global as well as cell-specific changes to infection which work in conjunction. This study has uncovered the substantial pathogen-specific effects on host epithelium, including many findings which were previously unknown in the absence of a single-cell approach.

The application of our understanding of host-microbiome interactions is especially relevant in a clinical context, where patient outcomes are often tied to their unique physiologies and immune responsiveness. Using the model of *ex vivo* infection of human peripheral blood mononuclear cells (PBMC) with *Salmonella*, one study performed scRNA-seq on unexposed and exposed cells to uncover the immune cell types and their subtypes before and after infection (Bossel Ben-Moshe et al., 2019; Figure 1H). Through this information, an algorithm was developed to deconvolute bulk measurements of PBMCs from pathogen exposures into cell types as well as subpopulations depending upon pathogen exposure. This was subsequently applied to bulk RNA-seq data from cohorts of tuberculosis (TB) patients at different stages of disease (Bossel Ben-Moshe et al., 2019). This algorithm was able to stratify latent TB infection patients at baseline (prior to presentation of active disease symptoms) into those who would progress to active disease and those who would not. This represents the striking power of scRNA-seq of host cells in the context of microbial infections, whereby data on monocyte infections, and their associated signatures, from one type of pathogen can be used and applied to other pathogen-based diseases. This has tremendous clinical value in the context of prognosis for infectious disease patients and broader disease applications that are modulated by the microbiome.

Hosts with viral infections have been studied similarly to intracellular bacterial infections, with novel insights into the involvement of particular cell types and pathways in the host response. One study developed a computational tool called Viral-Track to distinguish viral RNA in scRNA-seq data from virally-infected host cells (Liao et al., 2020). This is achieved through comprehensive mapping of scRNA-seq data onto databases of known viral genomes. As a result, cell types associated with viral infections can be profiled separately from uninfected bystander cell populations. In this study, Viral-Track successfully recognized infected cells of multiple *in vivo* mouse models of infections and human clinical samples of hepatitis B infection and detected host factors associated with viral replication. The authors also applied Viral-Track to study bronchoalveolar lavage samples from moderate and severe patients of COVID-19 (Wang et al., 2020). This analysis revealed the differential effect of the virus on immune cells of mild and severe patients. While mild patients exhibited alveolar macrophage enrichment, severe patients had accumulated neutrophils, inflammatory monocytes, macrophages, and a more naïve phenotype of CD4^+^ T cells (Liao et al., 2020). Inflammatory signatures were observed to distinguish severe phenotypes: inflammatory chemokine genes in SPP1^+^ monocytes and genes associated with hypoxia or oxidative stress were upregulated while MHC class II and type I IFN genes were downregulated. Alveolar macrophages also displayed a severity-associated upregulation of particular chemokines and cathepsins. Through analysis of host cells in the context of viral infections, this study showcases the broad applicability of single-cell sequencing for dissecting mechanisms of viral infections, including cellular and molecular signatures in virus-induced pathologies. Moreover, it highlights the tremendous value of reads not mapped to the host genome in traditional scRNA-seq. In a clinical context, the Viral-Track tool is an example of how scRNA-seq computational pipelines can serve as diagnostic tools, identify immune modulation in viral illness, and recognize co-infections.

Together, these studies identify novel axes of intercellular variability within host and pathogen transcriptomics that can not only be used to understand disease pathogenesis but may also predict disease outcomes as well (Penaranda and Hung, 2019). This work underscores the importance of single-cell analysis of host and microbial cells when characterizing a tissue, organ, or complex organism-wide manifestation of the host-microbiome relationship.

## Conclusion

Despite the nascence of technological advances in single-cell biological characterization of microbes and their host interactions, several common themes have emerged in the field. First, single microbial cells can be analyzed with high resolution through a variety of modalities (Figures 1A–E), many of which have been adapted from eukaryotic applications and optimized for microbial use. Therefore, it is likely that ongoing advances in human single-cell biology will continue to inspire and inform microbial technologies. Conversely, the unique challenges that single-cell studies impose upon microbial organisms—with low RNA and DNA content and diversity of cell membranes and walls—challenge and encourage current technologies to improve in sensitivity and reproducibility, which serves to benefit both microbe and multicellular organism studies (Figures 1F–H).

Alongside improvements in genomic, transcriptomic, and spatial resolution in the aforementioned studies, there are now emerging methods which are able to combine multiple types of analysis such that researchers do not need to sacrifice one type of analysis for another. These methods also open up avenues for simultaneous studies of both host and microbes at their interfaces (Figure 1I). Through such single-cell studies, great advancements have been made in appreciating the role of commensals in modulating host physiology and cell differentiation pathways. Through the application to host-pathogen interactions, these investigations reveal the influences of pathogen heterogeneity on host disease states, novel host responses to infections, and homeostatic changes at the cell and tissue level. These insights can be used to identify more sensitive and accurate biomarkers for clinical diagnosis, predict and monitor treatment outcomes, and leverage host-microbiome interactions for beneficial purposes.

Lastly, these technologies allow for new biological questions to be answered, such as how cooperative behavior between subgroups of microorganisms and their hosts can achieve successful coordinated outcomes ranging from nutrient absorption to pathogen clearance. Moreover, the division of labor among bacterial and host cells in these responses, which might otherwise be lost in bulk averages of cellular behavior in RNA-seq, can now be further investigated. The technologies reviewed here will inspire future studies aimed at clarifying several outstanding questions in the field such as: How do bacterial subpopulations cooperate to achieve population-level growth advantages? How do host cell subsets coordinate anti-microbial responses to maximize effectivity and to minimize tissue damage? What are the hierarchies of engagement in cellular subsets on both the host and the microbial side? Answering these questions may enable a new way of thinking about how to therapeutically target various cellular subsets in the future.

## Author Contributions

PS performed literature research and wrote the manuscript. CT guided literature research and edited the manuscript. All authors contributed to the article and approved the submitted version.

## Conflict of Interest

The authors declare that the research was conducted in the absence of any commercial or financial relationships that could be construed as a potential conflict of interest.
